# The Validity and Reproducibility of Dietary Non-enzymatic Antioxidant Capacity Estimated by Self-administered Food Frequency Questionnaires

**DOI:** 10.2188/jea.JE20170063

**Published:** 2018-10-05

**Authors:** Ikuko Kashino, Mauro Serafini, Junko Ishihara, Tetsuya Mizoue, Ayaka Sunami, Koutatsu Maruyama, Norie Sawada, Manami Inoue, Akiko Nanri, Kayo Kurotani, Shamima Akter, Motoki Iwasaki, Shoichiro Tsugane

**Affiliations:** 1 Department of Epidemiology and Prevention, Center for Clinical Sciences, National Center for Global Health and Medicine, Tokyo, Japan; 2 Functional Foods and Metabolic Stress Prevention Laboratory, Bioscience and Technology for Food, Agriculture and Environment, University of Teramo, Teramo, Italy; 3 Department of Nutrition Management, Sagami Women’s University, Kanagawa, Japan; 4 Epidemiology and Prevention Division, Research Center for Cancer Prevention and Screening, National Cancer Center, Tokyo, Japan; 5 Department of Public Health, Juntendo University Graduate School of Medicine, Tokyo, Japan; 6 AXA Department of Health and Human Security, Graduate School of Medicine, the University of Tokyo, Tokyo, Japan; 7 Department of Nutritional Education, National Institute of Health and Nutrition, Tokyo, Japan

**Keywords:** non-enzymatic antioxidant capacity, dietary assessment, human, FRAP, ORAC

## Abstract

**Background:**

High dietary non-enzymatic antioxidant capacity (NEAC) has been inversely related to the incidence of degenerative diseases. However, few studies have investigated the validity and reproducibility of dietary NEAC estimated from a food frequency questionnaire (FFQ). We assessed the validity and reproducibility of FFQ-based dietary NEAC against a dietary record (DR).

**Methods:**

Participants were 244 men and 253 women who completed a 28-day DR and FFQs. NEAC for each food item was estimated according to available databases of antioxidant capacity, as measured by ferric reducing-antioxidant power (FRAP), oxygen radical absorbance capacity (ORAC), and total radical-trapping antioxidant parameter (TRAP). Using Spearman’s rank correlation coefficients (CCs), we assessed the validity for dietary NEACs from a 28-day DR and a FFQ, and the reproducibility for them from two FFQs administered at a 1-year interval. Additionally, joint classification and the Bland-Altman method were applied to assess agreement between the two methods.

**Results:**

Regarding validation, deattenuated CCs for the energy-adjusted overall dietary NEACs between FFQ and DR for FRAP, ORAC, and TRAP were 0.52, 0.54, and 0.52, respectively, for all subjects. Extreme miscategorization rates by joint classification analysis were 2% for FRAP and ORAC and 1% for TRAP. Regarding reproducibility, CCs between the energy-adjusted dietary NEACs from two FFQs were 0.64 for FRAP and 0.65 for ORAC and TRAP.

**Conclusion:**

The validity and reproducibility of dietary NEAC of total food from the FFQ were moderate. Estimations of dietary NEAC using FFQ would be useful in studying disease relationships by categorizing habitual dietary NEAC.

## INTRODUCTION

The human diet contains a wide array of redox-active ingredients, such as vitamins C and E, as well as non-nutrient antioxidants, such as flavonoids, which efficiently modulate cellular antioxidant status and reduce oxidative stress. Dietary patterns and intake of foods rich in antioxidants have demonstrated inverse associations with oxidative stress-related chronic disease risks, including type 2 diabetes^1^ and cardiovascular disease (CVD).^2^ Moreover, antioxidants are reported to cooperatively reduce oxidative stress and risk of cancer^3^ and mortality^4^ through efficient cooperation between components of the redox network. Given the potential for synergistic interaction effects between various dietary and endogenous antioxidants, use of indicator to estimate the overall antioxidant effect of the diet would represent a valuable tool.^5^ Non-enzymatic antioxidant capacity (NEAC) measurements aim to assess the free radical-reducing capacity of antioxidants, as well as iron-reducing capacity, in consideration of the synergistic effect of antioxidants present in food and biological samples.^6^ Among the different methodologies, ferric reducing-antioxidant power (FRAP), oxygen radical absorbance capacity (ORAC), and total radical-trapping antioxidant parameter (TRAP) are established and validated assays for the measurement of NEAC in foods and biological fluids.^6^

A number of countries have established dietary NEAC databases of generally consumed foods.^7^^–^^11^ Several large cohort studies in Western countries have recently used these databases to estimate food frequency questionnaire (FFQ)-based dietary NEAC by summarizing the antioxidant capacity values of individual food items to estimate the intake of antioxidants from diet. These have reported inverse associations between FFQ-based dietary NEAC and the incidence of stroke,^12^ heart failure,^13^ cancer,^14^ and mortality.^15^ As FFQs can be simply and practically administered and analyzed for large numbers of people, they are often used to assess dietary intake in epidemiological studies, in contrast with multiple-day dietary records (DR), which directly measure details of individual intake. Thanks to these characteristics, FFQ-based dietary NEAC is now considered a convenient new epidemiological tool. However, it remains unclear whether FFQ-based dietary NEAC reflects true dietary antioxidant capacity. An Italian study of the validity of FFQ-based dietary NEAC against a DR reported only a moderate association,^16^ whereas a study in Swedish women that compared NEAC estimates from two FFQs completed 1 year apart reported that reproducibility was high.^17^ Given that FFQs are developed specifically for individual countries and regions, the validity and reproducibility of FFQ-based dietary NEAC must be verified in each country before using them in research.

Here, we aimed to examine the validity and reproducibility of Japanese FFQ-based dietary NEAC using data of the validation study from the Japan Public Health Center-based Prospective Study (JPHC study).

## METHODS

### JPHC Study procedure and subjects

The JPHC study, covering 11 Public Health Center areas nationwide, was launched in 1990 for Cohort I and in 1993 for Cohort II.^18^^,^^19^ Of these, we carried out the FFQ validity and reproducibility study in 10 areas, excluding Tokyo (Iwate, Akita, Nagano, and Okinawa-Chubu in cohort I and Ibaraki, Niigata, Kochi, Nagasaki, Okinawa-Miyako, and Osaka in cohort II). These were established in February 1994 and May 1996, respectively, as described elsewhere.^18^^,^^19^ In brief, a total of 247 participants (122 men and 125 women) for Cohort I and 392 participants (196 married couples) for Cohort II were initially registered in the study on a voluntary basis but not by random sampling of JPHC study participants. Of these, we excluded 142 participants who did not complete the two FFQs with a 1-year interval and a DR (14-day record in Okinawa-Chubu in Cohort I; and 28-day record in the other 9 areas in Cohort I and II). Finally, a total of 497 participants (244 men and 253 women; 209 participants in Cohort I and 288 participants in Cohort II) (78%) were available for analysis. Before starting the validity study, sample size calculation was done to detect the correlation coefficient of 0.25, which was observed in a previous study.^18^^,^^19^ The number of subjects required to detect this difference in correlation was approximately 112 (alpha = 0.05, beta = 0.20). Subjects from both cohorts were healthy volunteers without dietary restrictions who were not under- or overweight. Participants provided oral or written informed consent before the study. The study did not undergo ethical approval since it was conducted before the advent of ethical guidelines for epidemiology research in Japan, which mandate such approval.

### Dietary assessment

Data collection has been described in detail elsewhere.^19^^,^^20^ In brief, the participants completed the FFQ twice, at an approximately 1-year interval. The majority of the FFQ for the evaluation of validity (FFQ_V) were completed 3 months after the last DR (8 of 10 areas), while some of them were completed either with the last DR (1 area) or 6 months after the last DR (1 area). The FFQ for the evaluation of reproducibility (FFQ_R) was administered 1 year before or after the FFQ_V and was compared with the FFQ_V. The FFQ included questions on 138 food items (with standard portions/units and eating frequency) consumed during the previous year, as well as 14 supplementary questions regarding dietary and cooking behaviors and supplements. Composition values for 147 food items were developed from the responses.^21^

We collected 7-day DRs over four seasons (a total of 28 days), namely spring (May), summer (August), autumn (November), and winter (February), except in the Chubu public health center (PHC) area in Okinawa (two seasons). The survey method using DRs has been described elsewhere.^19^^,^^22^ In JPHC study cohort I and II, median correlation coefficients between food groups measured with the FFQ and DR were 0.38 and 0.41 for men, and 0.32 and 0.30 for women, respectively.^19^^,^^22^ Furthermore, correlation coefficients for food groups selected for this study, which were measured with the FFQ and DR, ranged from 0.22 for vegetables to 0.76 for alcoholic beverages among men in cohort I and from 0.15 for fungi to 0.55 for fruits among men in cohort II, while corresponding ranges were 0.15 for nuts and seeds to 0.50 for alcoholic beverages among women in cohort I and 0.12 for fungi to 0.49 for alcoholic beverages among women in cohort II, respectively.^19^^,^^22^

### Dietary NEAC levels

To estimate the FFQ- and DR-based dietary NEACs for each subject in Cohort I and II, we used published databases in which the NEAC of individual foods was analyzed in the same laboratory using FRAP, which measures the ability of antioxidant to reduce Fe^3+^ (ferric ion) to Fe^2+^ (ferrous iron), and TRAP, which measures the chain-breaking antioxidant capacity to scavenge peroxyl radicals.^8^^,^^9^ Moreover, we also selected ORAC, which is based on the same chemical principle as TRAP but which measures area under the curve of the radical-induced fluorescence decay.^7^^,^^10^^,^^11^^,^^23^^,^^24^ To avoid heterogeneity of measurement, we selected most of the foods (57 food items) from the largest published ORAC database^7^ and from an ORAC database of Japanese foods (36 food items).^11^ Additionally, to obtain values for foods available in the Japanese FFQ but not in the main ORAC database, we selected a few food items (8 food items) from other publications.^10^^,^^23^^,^^24^ If foods were not directly matched to databases, NEAC values were imputed using the following procedures: for dried foods, NEAC was calculated using the ratios of water specified in the Japanese food composition tables between dried and raw^25^; for Japanese pickled vegetables, were assigned the NEAC levels of the same raw vegetable; and when specific data for a Japanese food were not available, such as for navel oranges, were used data of the same food but with a different origin, such as for Valencia oranges.

Finally, we assigned the NEAC of 58 food items using FRAP, 55 using ORAC, and 51 using TRAP in the FFQ; and 161 food items using FRAP, 175 using ORAC, and 148 using TRAP in 464 items that possibly have antioxidant capacities in the DR. Overall dietary NEAC was calculated by multiplying the NEAC values of single foods by the amount of each food consumed, and then summing the NEAC levels of all foods for each subject. The food and beverage groups investigated in this study were selected from food groups with antioxidant capacities in published databases^7^^–^^11^^,^^23^^,^^24^ and formed on the basis of the Japan’s Standard Tables of Food Composition.^25^

### Statistical analysis

Descriptive data were expressed as means with standard deviations (SDs) for continuous variables or percentages for categorical variables. Dietary NEAC was adjusted for energy using the residual method in a regression model.^26^ To evaluate the trend association between dietary NEAC estimated in FFQ and characteristics, we conducted the Mantel-Haenszel chi-square test for categorical variables and linear regression analysis for continuous variables, with the ordinal numbers 1 to 3 assigned to each tertile category of dietary NEAC. The contribution of the NEAC of each food to the dietary NEAC was computed as: % NEAC food group = NEAC food group * 100/overall dietary NEAC. Validity of the FFQ using dietary NEAC levels from the DR was evaluated using Spearman’s rank correlation coefficients (CCs) for energy-unadjusted (crude), energy-adjusted, and deattenuated values, the latter of which were corrected for the attenuating effect of random intra-individual error (deattenuation). Deattenuation was performed using the following formula: deattenuated $\text{CCs}=r\times \sqrt{1+(\lambda_{\text{X}}/{\text{n}}_{\text{x}})}$, where *r* is the observed CC of energy-adjusted dietary NEAC, λ_X_ is the ratio of inter-individual to intra-individual variance for the DR, and n_x_ is the number of DRs for each subject.^27^ Additionally, the CCs for the dietary NEACs derived from the two FFQs administered 1 year apart were calculated to determine the reproducibility of the FFQ. We computed the CCs for the validity and reproducibility of each food and beverage group using the same formula. Furthermore, Bland-Altman analysis was performed, in which the mean agreement between the two dietary methods in estimating dietary NEAC was calculated. This method plotted mean intake from the two methods, (FFQ + DR)/2, on the x axis, and the difference between the two methods, FFQ − DR, on the y axis. Before plotting, the energy-adjusted dietary NEAC was log-transformed, as recommended by Bland and Altman,^28^^,^^29^ because dietary data often show proportional bias. As dietary NEAC was log-transformed, antilogging was necessary to interpret agreement. Mean agreement and limit of agreement (LOA: mean agreement ±2 SD) were expressed as a percentage, with 100% mean agreement indicating complete agreement. For example, a mean agreement of 150% indicated that on average, the FFQ estimates for dietary NEAC were 1.5 times the DR estimates. Overall agreement was assessed using the mean of the difference, width of LOA, and the dependence of difference on the magnitude of estimates test by fitting the regression line of differences. To assess the agreement of categorization, the energy-adjusted dietary NEACs derived from the FFQs and DRs were divided into quintiles, and the percentages of subjects classified into the same (agreement), the same or adjacent (adjacent agreement), and opposite categories (disagreement) were calculated using the joint classification method. Two-sided *P* values <0.05 were regarded as statistically significant. All analyses were performed using the SAS statistical software package, version 9.3 (SAS Institute Inc., Cary, NC, USA).

## RESULTS

Mean energy intake in all subjects was 2,027 (SD, 430) kcal for the DR, 2,123 (SD, 660) kcal for FFQ_R, and 2,043 (SD, 683) kcal for FFQ_V. Dietary NEACs estimated using the two FFQs and DR for men and women are shown in Table 1. The contribution of FFQ-based NEAC levels estimated using all measurement methods decreased in the order of beverages (green tea is more than 94%), fruits, and vegetables in energy-adjusted NEACs for men and women combined. The contribution of DR-based NEAC levels decreased in the order of beverages (green tea is more than 92%), vegetables, and fruits for FRAP and TRAP, and vegetables, beverages (green tea is more than 90%), and fruits for ORAC, in both energy-adjusted and -unadjusted NEAC for men and women combined. Similar tendencies were observed for men and women separately, except for ORAC in women (eTable 1 and eTable 2).

**Table 1.** Dietary antioxidant capacity of the food and beverage groups in men and women (*n* = 497)^a^

Food	FFQ_R^b^		Contribution (%)	FFQ_V^c^		Contribution (%)	28 day-DR^d^		Contribution (%)
		
	Mean	SD		Mean	SD		Mean	SD	
FRAP, µmol Fe^2+^/day									
Crude values									
Total food	15,918	9,929		15,118	9,340		8,879	4,598	
Cereals	391	311	2.5	371	290	2.5	222	164	2.5
Potatoes	78	73	0.5	77	80	0.5	85	55	1.0
Nuts and seeds	8	13	0.1	6	14	0.0	68	167	0.8
Vegetables	1,375	1,001	8.6	1,364	1,065	9.0	1,416	507	15.9
Fruits	2,188	2,113	13.7	2,254	2,259	14.9	904	588	10.2
Mushrooms	321	287	2.0	313	293	2.1	264	163	3.0
Confectioneries	85	145	0.5	78	135	0.5	35	82	0.4
Beverages	11,469	8,738	72.1	10,649	8,086	70.4	5,872	4,152	66.1
Green tea	11,036	8,757	69.3	10,153	8,126	67.2	5,430	4,111	61.2
Energy adjustment (residual method)									
Total food	15,644	9,096		14,798	8,800		8,861	4,549	
Cereals	374	262	2.4	361	270	2.4	213	142	2.4
Potatoes	76	67	0.5	73	67	0.5	84	51	0.9
Nuts and seeds	8	13	0.1	7	16	0.0	71	187	0.8
Vegetables	1,332	879	8.5	1,283	797	8.7	1,405	476	15.9
Fruits	2,051	1,540	13.1	2,072	1,500	14.0	904	587	10.2
Mushrooms	310	260	2.0	296	270	2.0	263	161	3.0
Confectioneries	83	136	0.5	75	119	0.5	38	99	0.4
Beverages	11,428	8,700	73.1	10,636	8,376	71.9	5,884	4,174	66.4
Green tea	11,163	9,204	71.4	10,339	9,051	69.9	5,440	4,112	61.4

ORAC, µmol TE/day									
Crude values									
Total food	8,854	5,034		8,668	5,259		5,935	2,289	
Cereals	449	537	5.1	418	513	4.8	132	242	2.2
Potatoes	232	194	2.6	229	228	2.6	247	131	4.2
Nuts and seeds	75	122	0.8	60	128	0.7	51	85	0.9
Vegetables	1,339	885	15.1	1,354	1,024	15.6	1,917	708	32.3
Fruits	3,110	2,837	35.1	3,199	3,109	36.9	1,553	1,057	26.2
Mushrooms	56	50	0.6	55	51	0.6	46	28	0.8
Confectioneries	143	245	1.6	133	229	1.5	96	238	1.6
Beverages	3,447	2,583	38.9	3,218	2,425	37.1	1,767	1,249	29.8
Green tea	3,249	2,578	36.7	2,989	2,393	34.5	1,599	1,210	26.9
Energy adjustment (residual method)									
Total food	8,588	3,958		8,318	4,040		5,918	2,231	
Cereals	447	598	5.2	572	1,097	6.9	133	246	2.2
Potatoes	225	173	2.6	211	169	2.5	247	129	4.2
Nuts and seeds	83	181	1.0	77	237	0.9	58	111	1.0
Vegetables	1,288	742	15.0	1,262	733	15.2	1,896	656	32.0
Fruits	2,933	2,128	34.2	2,956	2,149	35.5	1,553	1,054	26.2
Mushrooms	54	45	0.6	52	47	0.6	46	28	0.8
Confectioneries	140	232	1.6	127	203	1.5	102	273	1.7
Beverages	3,426	2,514	39.9	3,233	2,564	38.9	1,768	1,243	29.9
Green tea	3,277	2,679	38.2	3,032	2,626	36.5	1,601	1,210	27.1

TRAP, µmol TE/day									
Crude values									
Total food	6,290	4,117		5,930	3,865		3,469	1,932	
Cereals	58	63	0.9	54	59	0.9	25	33	0.7
Potatoes	18	17	0.3	18	18	0.3	20	13	0.6
Nuts and seeds	1	2	0.0	1	2	0.0	5	11	0.1
Vegetables	522	363	8.3	503	377	8.5	576	193	16.6
Fruits	764	723	12.1	783	784	13.2	357	231	10.3
Mushrooms	122	110	1.9	120	112	2.0	101	62	2.9
Confectioneries	23	40	0.4	22	37	0.4	12	27	0.3
Beverages	4,779	3,721	76.0	4,428	3,447	74.7	2,371	1,768	68.3
Green tea	4,678	3,712	74.4	4,304	3,444	72.6	2,302	1,742	66.4
Energy adjustment (residual method)									
Total food	6,194	3,824		5,821	3,718		3,466	1,919	
Cereals	55	59	0.9	53	61	0.9	24	31	0.7
Potatoes	18	15	0.3	17	15	0.3	20	12	0.6
Nuts and seeds	1	2	0.0	1	2	0.0	5	12	0.1
Vegetables	504	312	8.1	473	284	8.1	570	176	16.4
Fruits	717	526	11.6	720	519	12.4	357	231	10.3
Mushrooms	118	99	1.9	113	103	1.9	100	61	2.9
Confectioneries	23	37	0.4	20	33	0.3	12	31	0.3
Beverages	4,763	3,691	76.9	4,470	3,720	76.8	2,373	1,764	68.5
Green tea	4,722	3,870	76.2	4,370	3,797	75.1	2,306	1,742	66.5

FFQ, food frequency questionnaire; DR, dietary record; SD, standard deviation; TE, trolox equivalent; FRAP, ferric reducing-antioxidant power; ORAC, oxygen radical absorbance capacity; TRAP, total radical-trapping antioxidant parameter.

^a^NEAC levels of 58 food items by FRAP, 55 by ORAC, and 51 by TRAP in the FFQ are assigned.

^b^FFQ_R was administered 1 year after or before FFQ_V.

^c^FFQ_V was administered 1 year after completion of the DRs.

^d^DR was collected over a 1-year period.

Table 2 presents subject characteristics by tertile of dietary NEAC estimated with the FFQ. Subjects with a higher FFQ-based dietary NEAC for all measurements were more likely to be older, women, and non-current smokers. The FFQ-based dietary NEAC for all measurements increased with increases in the intake of vitamin C, α- and β-carotene, cryptoxanthin, and a-tocopherol.

**Table 2.** Characteristics of study subjects by tertile of dietary NEAC estimated in the FFQ

Variables		FRAP							ORAP							TRAP						
		
		T1 (low)		T2		T3 (high)		Trend *P*^a^	T1 (low)		T2		T3 (high)		Trend *P*^a^	T1 (low)		T2		T3 (high)		Trend *P*^a^
								
		Mean	SD	Mean	SD	Mean	SD		Mean	SD	Mean	SD	Mean	SD		Mean	SD	Mean	SD	Mean	SD	
Age	years	54.4	6.6	56.9	6.5	57.0	6.8	<0.01	54.5	6.4	56.5	6.8	57.3	6.8	<0.01	54.4	6.6	56.5	6.5	57.3	6.8	<0.01
Sex, men	(%)	55.8		54.2		37.4		<0.01	64.9		49.4		33.1		<0.01	57.0		51.2		39.2		<0.01
BMI^b^	kg/m^2^	23.8	3	24.8	14	25.1	18.2	0.67	24.0	3.0	23.7	2.9	26.0	22.7	0.23	23.9	3	24.7	14	25.0	18.2	0.71
Total Physical activity	MET/hour/week	33.0	6.4	32.5	6	32.5	5.4	0.65	33.2	6.6	31.9	5.4	32.9	5.8	0.16	33.0	6.4	32.6	6.1	32.5	5.3	0.71
Current smoker	(%)	19.6		15.7		11.6		0.05	22.0		14.6		10.4		<0.01	20.3		15.7		11		0.02
Total energy intake	kcal/day	2,040	621	2,103	747	1,998	659	0.36	2,032	594	2,031	605	2,078	815	0.77	2,040	624	2,125	743	1,976	657	0.13
α-carotene^c^	µg/day	613	500	666	540	907	799	<0.01	590	513	701	513	895	810	<0.01	604	498	676	538	906	802	<0.01
β-carotene^c^	µg/day	3,575	1,949	4,129	2,281	5,412	3,183	<0.01	3,436	2,014	4,131	2,023	5,547	3,232	<0.01	3,607	1,964	4,162	2,286	5,346	3,201	<0.01
Cryptoxanthin^c^	µg/day	1,071	1,082	1,361	1,321	1,611	1,246	<0.01	745	540	1,216	964	2,080	1,571	<0.01	1,118	1,165	1,351	1,260	1,575	1,252	<0.01
Vitamin C^c^	mg/day	112	44	152	54	216	79	<0.01	101	35	147	34	231	74	<0.01	114	47	153	53	214	81	<0.01
α-tocopherol^c^	mg/day	6.4	2.0	6.9	2.2	7.6	2.2	<0.01	6.2	2.1	6.7	1.8	7.9	2.3	<0.01	6.3	1.9	7.0	2.2	7.5	2.2	<0.01

BMI, body mass index; MET, metabolic equivalent; T, tertile.

^a^Mantel-Haenszel chi-square test was used for categorical variables and linear regression analysis for continuous variables.

^b^BMI was calculated as body weight (kilograms) divided by the square of body height (meters).

^c^Energy adjustment was performed according to the residual method.

Table 3 shows CCs between dietary NEACs estimated using the FFQ and DR. Deattenuated CCs for energy-adjusted NEACs of total food for FRAP, ORAC, and TRAP were 0.52, 0.54, and 0.52 overall, 0.46, 0.53, and 0.47 for men, and 0.54, 0.49, and 0.53 for women, respectively. For all subjects, deattenuated CCs of energy-adjusted dietary NEACs derived from the FFQ and DR for FRAP, ORAC, and TRAP ranged from 0.21 for nuts and seeds to 0.57 for fruits, 0.14 for cereals to 0.57 for fruits, and 0.21 for nuts and seeds to 0.59 for fruits, respectively. In men, the CCs for FRAP, ORAC, and TRAP ranged from 0.12 for nuts and seeds to 0.61 for fruits, 0.16 for cereals to 0.62 for fruits, and 0.13 for nuts and seeds to 0.62 for fruits, respectively. In women, the CCs for FRAP, ORAC, and TRAP ranged from 0.24 for nuts and seeds to 0.55 for beverages and green tea, 0.19 for cereals to 0.55 for green tea, and 0.24 for nuts and seeds to 0.55 for beverages and green tea, respectively.

**Table 3.** Ranking validity of FFQ-based NEAC of foods and beverages by comparison to 28-day DR

Food	FRAP			ORAC			TRAP		
		
	Spearman’s correlation coefficient			Spearman’s correlation coefficient			Spearman’s correlation coefficient		
		
	Crude	Energy adjustment	Deattenuated	Crude	Energy adjustment	Deattenuated	Crude	Energy adjustment	Deattenuated
All									
Total food	0.49	0.51	0.52	0.50	0.53	0.54	0.50	0.51	0.52
Cereals	0.39	0.31	0.33	0.20	0.13	0.14	0.30	0.28	0.30
Potatoes	0.24	0.30	0.32	0.28	0.33	0.35	0.24	0.30	0.32
Nuts and seeds	0.26	0.19	0.21	0.31	0.23	0.25	0.27	0.19	0.21
Vegetables	0.32	0.35	0.36	0.32	0.32	0.34	0.32	0.37	0.38
Fruits	0.54	0.55	0.57	0.54	0.55	0.57	0.55	0.57	0.59
Mushrooms	0.27	0.33	0.35	0.27	0.33	0.35	0.27	0.33	0.35
Confectioneries	0.33	0.21	0.23	0.33	0.22	0.24	0.33	0.21	0.23
Beverages	0.48	0.48	0.48	0.46	0.48	0.48	0.49	0.49	0.50
Green tea	0.50	0.49	0.50	0.50	0.49	0.52	0.50	0.49	0.50
Men									
Total food	0.45	0.46	0.46	0.52	0.52	0.53	0.47	0.47	0.47
Cereals	0.39	0.35	0.37	0.22	0.15	0.16	0.31	0.29	0.31
Potatoes	0.23	0.31	0.32	0.26	0.34	0.36	0.23	0.31	0.32
Nuts and seeds	0.26	0.11	0.12	0.35	0.23	0.25	0.26	0.12	0.13
Vegetables	0.31	0.31	0.32	0.32	0.27	0.29	0.30	0.31	0.32
Fruits	0.59	0.59	0.61	0.59	0.60	0.62	0.60	0.60	0.62
Mushrooms	0.26	0.30	0.32	0.26	0.31	0.33	0.26	0.30	0.32
Confectioneries	0.32	0.34	0.37	0.32	0.19	0.21	0.32	0.34	0.37
Beverages	0.41	0.39	0.39	0.41	0.41	0.41	0.44	0.40	0.40
Green tea	0.45	0.40	0.40	0.45	0.40	0.40	0.45	0.40	0.40
Women									
Total food	0.52	0.53	0.54	0.45	0.48	0.49	0.52	0.53	0.53
Cereals	0.35	0.26	0.28	0.18	0.18	0.19	0.28	0.25	0.27
Potatoes	0.26	0.27	0.29	0.30	0.32	0.34	0.26	0.27	0.29
Nuts and seeds	0.27	0.22	0.24	0.27	0.21	0.23	0.28	0.22	0.24
Vegetables	0.35	0.36	0.37	0.35	0.33	0.35	0.37	0.38	0.39
Fruits	0.44	0.40	0.42	0.45	0.39	0.41	0.45	0.41	0.43
Mushrooms	0.29	0.36	0.38	0.29	0.36	0.38	0.29	0.36	0.38
Confectioneries	0.29	0.36	0.39	0.29	0.36	0.39	0.28	0.35	0.38
Beverages	0.54	0.54	0.55	0.51	0.52	0.53	0.54	0.54	0.55
Green tea	0.54	0.54	0.55	0.54	0.54	0.55	0.54	0.54	0.55

FFQ, food frequency questionnaire; DR, dietary record; SD, standard deviation; FRAP, ferric reducing-antioxidant power; ORAC, oxygen radical absorbance capacity; TRAP, total radical-trapping antioxidant parameter.

Reproducibility of dietary NEAC between two FFQs administered at a 1-year interval is presented in Table 4. The CCs for energy-adjusted NEACs of total food for FRAP, ORAC, and TRAP were 0.64, 0.65, and 0.65 overall, 0.57, 0.59, and 0.59 for men, and 0.67, 0.59, and 0.67 for women, respectively. In individual food groups, the CCs of energy-adjusted NEAC between two FFQs for FRAP, ORAC, and TRAP ranged from 0.43 for nuts and seeds to 0.61 for fruits and vegetables, from 0.43 for nuts and seeds to 0.64 for fruits, and 0.44 for nuts and seeds to 0.64 for vegetables overall. The CCs for FRAP, ORAC, and TRAP ranged from 0.39 for nuts and seeds to 0.54 for fruits, 0.38 for nuts and seeds to 0.58 for fruits, and 0.41 for nuts and seeds to 0.56 for fruits and vegetables in men, and from 0.44, 0.43, and 0.45 for nuts and seeds to 0.66, 0.67, and 0.67 for beverages in women.

**Table 4.** Reproducibility of the FFQ-based NEAC of foods and beverages by FFQ_R^a^ administered 1 year after and before FFQ_V^b^

Food	FRAP		ORAC		TRAP	
		
	Spearman’s correlation coefficients		Spearman’s correlation coefficients		Spearman’s correlation coefficients	
		
	Crude	Energy adjustment	Crude	Energy adjustment	Crude	Energy adjustment
All						
Total food	0.68	0.64	0.67	0.65	0.69	0.65
Cereals	0.55	0.55	0.52	0.52	0.53	0.54
Potatoes	0.56	0.53	0.60	0.56	0.56	0.53
Nuts and seeds	0.57	0.43	0.57	0.43	0.57	0.44
Vegetables	0.63	0.61	0.65	0.61	0.66	0.64
Fruits	0.63	0.61	0.66	0.64	0.64	0.62
Mushrooms	0.53	0.53	0.53	0.53	0.53	0.53
Confectioneries	0.60	0.58	0.60	0.58	0.60	0.58
Beverages	0.64	0.60	0.64	0.61	0.66	0.61
Green tea	0.66	0.60	0.66	0.61	0.66	0.60
Men						
Total food	0.64	0.57	0.68	0.59	0.66	0.59
Cereals	0.53	0.50	0.48	0.48	0.51	0.51
Potatoes	0.51	0.45	0.55	0.47	0.51	0.46
Nuts and seeds	0.56	0.39	0.56	0.38	0.56	0.41
Vegetables	0.60	0.53	0.63	0.51	0.65	0.56
Fruits	0.60	0.54	0.64	0.58	0.62	0.56
Mushrooms	0.52	0.47	0.52	0.47	0.52	0.47
Confectioneries	0.59	0.53	0.59	0.53	0.59	0.53
Beverages	0.58	0.51	0.59	0.52	0.62	0.51
Green tea	0.61	0.50	0.61	0.50	0.61	0.50
Women						
Total food	0.70	0.67	0.65	0.59	0.71	0.67
Cereals	0.55	0.55	0.55	0.54	0.56	0.56
Potatoes	0.59	0.52	0.62	0.55	0.59	0.52
Nuts and seeds	0.56	0.44	0.56	0.43	0.56	0.45
Vegetables	0.66	0.59	0.64	0.59	0.67	0.61
Fruits	0.62	0.53	0.64	0.56	0.62	0.53
Mushrooms	0.53	0.52	0.53	0.52	0.53	0.52
Confectioneries	0.58	0.53	0.58	0.53	0.58	0.53
Beverages	0.71	0.66	0.69	0.67	0.70	0.67
Green tea	0.70	0.66	0.70	0.66	0.70	0.66

DR, dietary record; FFQ, food frequency questionnaire; SD, standard deviation; FRAP, ferric reducing-antioxidant power; ORAC, oxygen radical absorbance capacity; TRAP, total radical-trapping antioxidant parameter.

^a^FFQ_R was administered 1 year before and after FFQ_V.

^b^FFQ_V was administered 1 year after completion of the DRs.

Additionally, we conducted a sensitivity analysis after excluding the participants (*n* = 113) from Okinawa, who might have had different food habits from other participants. As a result, we did not observe remarkable differences of those CCs of the validity and reproducibility before and after excluding Okinawa’s participants (data not shown).

**Figure 1.**
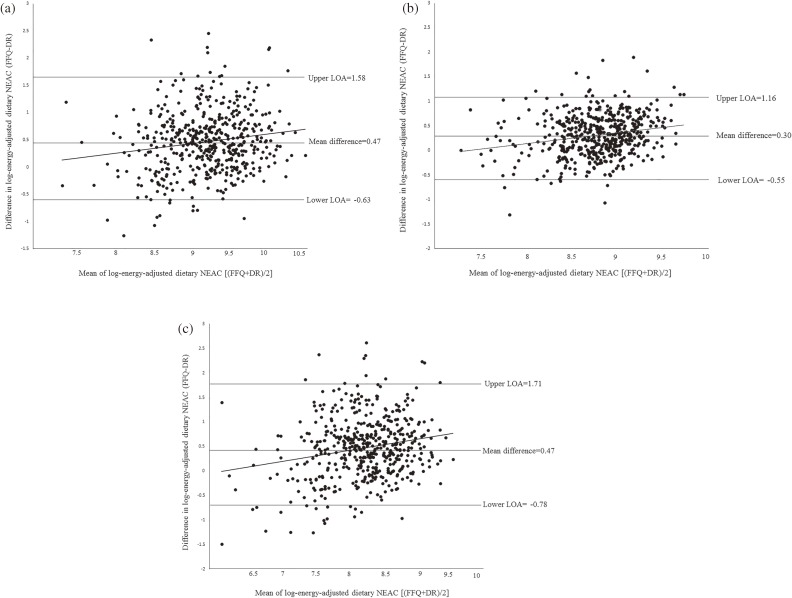
Bland-Altman method of assessing agreement between the FFQ and 28-day DR for energy-adjusted dietary NEAC in (a) FRAP (y = 0.1831x − 1.2309; *P* < 0.01), (b) ORAC (y = 0.2209x − 1.6309; *P* < 0.01), and (c) TRAP (y = 0.2391x − 1.5024; *P* < 0.01). DR, dietary record; FFQ, food frequency questionnaire; LOA, limit of agreement.

Agreement between the FFQ and 28-day DR for both sexes using the Bland-Altman plot is presented in Figure 1. FFQ estimates for overall dietary NEAC for FRAP, ORAC, and TRAP were 1.6 times, 1.4 times, and 1.6 times their DR estimates, respectively. Furthermore, 95% of all subjects’ FFQ estimates for FRAP, ORAC, and TRAP were between 0.5 and 4.9 times, 0.6 and 3.2 times, and 0.5 and 5.5 times the DR estimates, respectively. The fitted regression line of agreement indicated a significant linear trend. That is, a dependency existed between the difference in the two methods and the average of the two methods: as the dietary NEAC of individuals increased, so did the magnitude of the error between the FFQ and 28-day DR. Regarding the agreement of classification for the overall dietary NEAC using FFQ and DR, the proportion of subjects classified into the opposite extreme category was 2% for FRAP and ORAC, and 1% for TRAP in all subjects, while that of subjects classified into the same or an adjacent category was 73% for FRAP and TRAP and 72% for ORAC. The proportion of categorization in the same or adjacent category for men was 71% for all measurements, and that in the opposite extreme category was 2% for FRAP and TRAP and 1% for ORAC, while the proportion in the same or adjacent category for women was 72% for FRAP and TRAP and 70% for ORAC, and that in the opposite extreme category was 2% for FRAP and TRAP and 1% for ORAC measurements.

## DISCUSSION

In this study, we evaluated the validity of dietary NEAC measures between an FFQ and 28-day DR, and the reproducibility of NEAC on repeated administration of the FFQ at a 1-year interval. The validity and reproducibility of dietary NEAC of total food derived from the FFQ were moderate. However, the FFQ-based dietary NEAC tended to be overestimated compared to that from the DR, and agreement between the two methods decreased significantly as dietary NEAC increased. On the other hand, the FFQ-based dietary NEAC was suitable for categorizing subjects by individual dietary NEAC levels. This is the first study to evaluate the validity and reproducibility of Japanese FFQ-based dietary NEAC among Japanese.

We observed moderate validity for ranking individuals by dietary NEAC, with CCs between the FFQ-based energy-adjusted overall dietary NEAC and 28-day DR-based energy-adjusted dietary NEAC of 0.51 for FRAP, 0.53 for ORAC, and 0.51 for TRAP. These values were similar to those of previous studies, which showed moderate correlation between dietary NEACs derived from an FFQ and 3-day DR (*r* = 0.58 for TRAP and *r* = 0.52 for FRAP) in healthy Italian adults^16^ and between FFQ and 24-hour recall (HR) estimations of dietary NEAC in healthy Spanish adults (*r* = 0.62 for FRAP and *r* = 0.71 for ORAC).^30^ Our 28-day DR, which collected 7-day DRs four times over a single year, was clearly more suitable than a 3-day DR and 24-hour HR, which capture short-term diet, for estimating the dietary NEAC over 1 year.^31^ We, therefore, consider that our validation results are acceptable for ranking individuals by estimated values. Regarding the results by food groups, the previous Spanish study reported that CCs of NEAC for fruits/juice and vegetables were moderate, while those of NEAC from cereals and nuts were low.^30^ These results were similar to our present results. The validation levels of NEAC for nuts and seeds by all measurements and for cereals by ORAC might have been low overall and in men and women separately because we could not assign NEAC values to sufficient numbers of nuts and seeds using any of the measurements and of cereals using ORAC with the FFQ than with the DR (nuts and seeds: 1 item using all measurements in FFQ vs 7 items using FRAP and TRAP and 13 items using ORAC in DR; cereals using ORAC: 1 item in FFQ vs 9 items in DR). Regarding the NEAC of cereals using FRAP and TRAP, as we could assign NEAC levels to frequently consumed foods, such as rice, in both the FFQ and DR, the validation of FRAP and TRAP was considered to be moderate. Considering these low validation results, it would be difficult to estimate the FFQ-based antioxidant capacities of cereals using ORAC, and of nuts and seeds using any of the measurements.

FFQ-based dietary NEAC tended to be overestimated compared with DR-based NEAC, and agreement between them decreased significantly as dietary NEAC increased. Nevertheless, FFQ-based dietary NEAC was adequate for classifying subjects’ dietary NEACs: extreme miscategorization was only 1% to 2% for all measurements overall and in men and women separately. Results from our previous validation studies with same population showed clear overestimation of intakes with our FFQs compared with those with the DR, particularly for fruits and beverages.^19^^,^^22^^,^^32^ As NEAC derived from an FFQ and DR was estimated by multiplying the intake of food items and antioxidant capacity of food items, overestimation of food intake using an FFQ might have led to the overestimation of FFQ-based dietary NEAC compared with DR-based dietary NEAC. Additionally, the slope of the regression line of differences was positive, meaning that the FFQ-based NEAC intake was increasingly overestimated as overall NEAC intake increased. These results indicate that dose-response relationship associations between FFQ-based dietary NEAC and risk of disease might be overestimated, and should, therefore, be interpreted with caution. On the other hand, we observed adequate results for classifying subjects by dietary NEAC: percentages of subjects classified into the same or adjacent and opposite quartiles using the two methods ranged from 70% to 73% and 1% to 2% for all measurements, respectively. Epidemiological studies often analyze disease risk by categorizing subjects by the amount of food intake. These results therefore suggest that estimations of dietary NEAC using FFQ would be useful in studying disease relationships by categorizing habitual dietary NEAC.

Regarding the reproducibility of dietary NEAC of total foods using two FFQs conducted at a 1-year interval, CCs between the dietary NEACs of total food derived from two FFQs for FRAP, ORAC, and TRAP were 0.64, 0.65, and 0.65 for all subjects; 0.57, 0.59, and 0.59 for men; and 0.67, 0.59, and 0.67 for women, respectively. These CCs were of the same magnitude to those in a Swedish mammography cohort,^17^ which reported CCs for total food of 0.68 for FRAP, 0.65 for ORAC, and 0.71 for TRAP in women. Additionally, our CCs for the vegetable and fruit groups were also similar to those of the Swedish study in women (vegetables and fruits: *r* = 0.59 and 0.53 for FRAP, *r* = 0.59 and *r* = 0.56 for ORAC, and 0.61 and 0.53 for TRAP in women in our study vs *r* = 0.61 and 0.55 for FRAP, *r* = 0.58 and *r* = 0.56 for ORAC, and *r* = 0.59 and *r* = 0.56 for TRAP in the Swedish study). We, therefore, consider that the reproducibility of dietary NEAC in our study was acceptable.

We observed that the NEAC of beverages was the highest among all food items. The main contributor to beverage NEAC was Japanese green tea, at about 95%. Green tea was followed by fruits and vegetables in the sample overall and in men and women. Two previous studies among young (aged 18–22 years)^33^ and older Japanese women (65 years and older)^34^ also reported that the highest contributor to NEAC was beverages, such as green tea, followed by vegetables and fruits.^33^^,^^34^ Previous studies in Western countries reported that major contributors to dietary NEAC were fruits and vegetables, cereals, tea, and wine,^13^^,^^17^^,^^35^ although not all the beverage NEACs were described. These findings showed that the major common sources of dietary NEAC in Japan and Western countries are vegetables and fruits. In contrast, green tea in the Japanese diet and cereals, tea, and wine in Western countries were specific contributors to dietary NEAC in their respective regions.

Strengths of our study include its large number of subjects from multiple areas across Japan and the use of detailed records over the four seasons (total of 28 days), except in one area. Our study also has several limitations. First, although we could not use a Japanese NEAC database for FRAP, TRAP, and most of the ORAC foods and assign dietary NEAC to many food items due to the lack of information in the literature, we selected other countries’ databases analyzed by the same laboratory to maintain the homogeneity and reliability of analyses. Second, we did not measure blood NEAC for validity. Plasma NEAC is influenced by many factors, including endogenous antioxidants, which control homeostatic mechanisms of plasma antioxidants.^36^ For example, uric acid, an endogenous antioxidant from dietary purines, can provide as much as 60% of oxygen and free-radical scavenging in human serum and is highly correlated with plasma NEAC.^37^ Therefore, blood NEAC may not be suitable for validating FFQ-based NEAC, because blood antioxidant capacity is largely affected by endogenous antioxidants. Finally, as participants completed the FFQ after the DR, participant recall of dietary intake might have been influenced by administration of the DR. However, the reproducibility of dietary NEAC estimated between two FFQs administered at a 1-year interval was relatively high in our study, indicating that any influence of participant recall of dietary intake would be minor.

In conclusion, we found that the validity and reproducibility of the dietary NEAC of total food derived from an FFQ were acceptable. FFQ-based dietary NEAC was suitable for categorizing subjects by individual dietary NEAC. These estimates of the validity and reproducibility of FFQ-based NEAC can be used in interpreting the results of association studies in the JPHC Study.

## APPENDIX A. SUPPLEMENTARY DATA

eTable 1. Dietary antioxidant capacity of the food and beverage groups in men (n=244)

eTable 2. Dietary antioxidant capacity of the food and beverage groups in women (n=253)
