# Supplementary material for: The Validity and Reproducibility of Dietary Non-enzymatic Antioxidant Capacity Estimated by Self-administered Food Frequency Questionnaires
Source: J Epidemiol. 2018 Oct 5;28(10):428–36. doi: 10.2188/jea.JE20170063 (PMC6143376; doi:10.2188/jea.JE20170063)
Supplement: Supplementary file 1 [file je-28-428-s001.pdf]

eTable 1. Dietary antioxidant capacity of the food and beverage groups in men (n=244)

	FFQ _R[a]		Contribution	FFQ _V[b]		Contribution	28day-DR[c]		Contribution
Food	Mean	SD	(%)	Mean	SD	(%)	Mean	SD	(%)
FRAP, μmol Fe^{2+}/d									
Crude values									
Total food	15,753	9,823		14,591	8,526		9,013	4,741	
Cereals	444	361	2.8	405	316	2.8	264	191	2.9
Potatoes	73	75	0.5	71	75	0.5	89	62	1.0
Nuts and seeds	9	15	0.1	6	9	0.0	58	152	0.6
Vegetables	1,243	830	7.9	1,261	941	8.6	1,459	514	16.2
Fruits	1,840	1,549	11.7	1,933	1,798	13.2	736	529	8.2
Mushrooms	298	290	1.9	285	259	2.0	272	172	3.0
Confectioneries	65	112	0.4	62	119	0.4	24	61	0.3
Beverages	11,778	8,964	74.8	10,565	7,638	72.4	6,098	4,253	67.7
Green tea	11,184	9,031	71.0	9,856	7,738	67.5	5,417	4,232	60.1
Energy adjustment (residual method)									
Total food	15,418	9,203		14,212	7,825		8,978	4,593	
Cereals	425	315	2.8	396	311	2.8	257	181	2.9
Potatoes	71	66	0.5	66	65	0.5	88	57	1.0
Nuts and seeds	8	15	0.1	7	15	0.0	62	197	0.7
Vegetables	1,182	659	7.7	1,185	743	8.3	1,445	464	16.1
Fruits	1,725	1,342	11.2	1,796	1,286	12.6	734	526	8.2
Mushrooms	294	307	1.9	266	236	1.9	272	171	3.0

Confectioneries	69	145	0.4	61	104	0.4	24	60	0.3
Beverages	11,784	9,202	76.4	10,652	8,302	75.0	6,090	4,159	67.8
Green tea	12,562	13,302	81.5	10,592	10,581	74.5	5,429	4,167	60.5
ORAC, μmol TE/d									
Crude values									
Total food	8,343	4,570		8,053	4,532		5,764	2,420	
Cereals	519	636	6.2	448	554	5.6	165	292	2.9
Potatoes	215	201	2.6	200	197	2.5	246	138	4.3
Nuts and seeds	85	139	1.0	58	86	0.7	53	94	0.9
Vegetables	1,258	873	15.1	1,281	917	15.9	2,002	748	34.7
Fruits	2,617	2,102	31.4	2,757	2,544	34.2	1,291	978	22.4
Mushrooms	52	51	0.6	50	45	0.6	47	30	0.8
Confectioneries	110	190	1.3	105	202	1.3	64	181	1.1
Beverages	3,485	2,682	41.8	3,151	2,334	39.1	1,766	1,323	30.6
Green tea	3,293	2,659	39.5	2,902	2,279	36.0	1,595	1,246	27.7
Energy adjustment (residual method)									
Total food	8,055	3,841		7,725	3,607		5,731	2,304	
Cereals	541	866	6.7	803	2,002	10.4	166	291	2.9
Potatoes	210	180	2.6	184	158	2.4	246	135	4.3
Nuts and seeds	87	166	1.1	94	277	1.2	69	165	1.2
Vegetables	1,184	674	14.7	1,189	759	15.4	1,976	678	34.5
Fruits	2,468	1,845	30.6	2,570	1,902	33.3	1,282	952	22.4

Mushrooms	50	50	0.6	46	40	0.6	47	30	0.8
Confectioneries	118	254	1.5	104	179	1.3	64	181	1.1
Beverages	3,480	2,706	43.2	3,269	2,848	42.3	1,769	1,306	30.9
Green tea	3,621	3,689	45.0	3,072	2,964	39.8	1,598	1,226	27.9
TRAP, µmol TE/d									
Crude values									
Total food	6,175	4,130		5,650	3,568		3,426	3,718	
Cereals	66	73	1.1	58	64	1.0	30	61	0.9
Potatoes	17	17	0.3	16	17	0.3	21	15	0.6
Nuts and seeds	1	2	0.0	1	1	0.0	4	2	0.1
Vegetables	480	325	7.8	471	336	8.3	601	284	17.5
Fruits	643	528	10.4	670	612	11.9	292	519	8.5
Mushrooms	114	111	1.8	109	99	1.9	104	103	3.0
Confectioneries	18	31	0.3	17	33	0.3	8	33	0.2
Beverages	4,835	3,836	78.3	4,308	3,276	76.2	2,363	3,720	69.0
Green tea	4,741	3,828	76.8	4,178	3,280	73.9	2,296	1,794	67.0
Energy adjustment (residual method)									
Total food	6,057	3,925		5,514	3,388		3,417	1,962	
Cereals	65	81	1.1	57	70	1.0	29	42	0.8
Potatoes	16	15	0.3	15	15	0.3	20	13	0.6
Nuts and seeds	1	2	0.0	1	1	0.0	4	11	0.1
Vegetables	454	250	7.5	442	288	8.0	595	175	17.4

Fruits	602	456	9.9	623	441	11.3	291	204	8.5
Mushrooms	111	112	1.8	101	89	1.8	104	65	3.0
Confectioneries	18	36	0.3	16	28	0.3	8	20	0.2
Beverages	5,008	4,416	82.7	4,608	4,453	83.6	2,366	1,797	69.2
Green tea	5,245	5,405	86.6	4,441	4,330	80.5	2,300	1,765	67.3

FFQ, food frequency questionnaire; DR, dietary record; SD, standard deviation; TE, trolox quivalent; FRAP, ferric reducing-antioxidant power; ORAC, oxygen radical absorbance capacity; TRAP, total radical-trapping antioxidant parameter.

[a] FFQ_R was administered 1 year after and before FFQ_V.

[b] FFQ_V was administered 1 year after completion of the DRs.

[c] DR was collected over a 1-year period.

eTable 2. Dietary antioxidant capacity of the food and beverage groups in women (n=253)

Food	FFQ _R[a]		Contribution	FFQ _V[b]		Contribution	28day-DR[c]		Contribution
	Mean	SD	(%)	Mean	SD	(%)	Mean	SD	(%)
FRAP, μmol Fe^{2+}/d									
Crude values									
Total food	16,077	10,047		15,627	10,054		8,751	4,461	
Cereals	340	243	2.1	339	259	2.2	182	121	2.1
Potatoes	84	70	0.5	84	84	0.5	81	48	0.9
Nuts and seeds	7	11	0.0	7	17	0.0	76	181	0.9
Vegetables	1,503	1,129	9.3	1,464	1,166	9.4	1,375	497	15.7
Fruits	2,523	2,498	15.7	2,564	2,595	16.4	1,065	598	12.2
Mushrooms	342	283	2.1	341	321	2.2	256	152	2.9
Confectioneries	104	168	0.6	94	148	0.6	46	97	0.5
Beverages	11,171	8,523	69.5	10,731	8,509	68.7	5,655	4,049	64.6
Green tea	10,893	8,499	67.8	10,439	8,488	66.8	5,442	3,999	62.2
Energy adjustment (residual method)									
Total food	15,793	8,913		15,240	9,292		8,740	4,498	
Cereals	329	214	2.1	330	236	2.2	177	112	2.0
Potatoes	81	64	0.5	78	65	0.5	81	45	0.9
Nuts and seeds	7	14	0.0	6	14	0.0	82	248	0.9
Vegetables	1,450	957	9.2	1,348	762	8.8	1,362	469	15.6
Fruits	2,282	1,486	14.4	2,250	1,447	14.8	1,039	551	11.9

Mushrooms	333	256	2.1	318	282	2.1	254	153	2.9
Confectioneries	108	191	0.7	94	151	0.6	44	85	0.5
Beverages	11,104	8,272	70.3	10,674	8,519	70.0	5,688	4,147	65.1
Green tea	10,853	8,344	68.7	10,403	8,596	68.3	5,477	4,101	62.7
ORAC, μmol TE/d									
Crude values									
Total food	9,346	5,407		9,260	5,824		6,100	2,148	
Cereals	381	410	4.1	388	469	4.2	100	176	1.6
Potatoes	248	186	2.7	257	252	2.8	248	124	4.1
Nuts and seeds	65	101	0.7	62	158	0.7	50	74	0.8
Vegetables	1,418	891	15.2	1,425	1,115	15.4	1,835	658	30.1
Fruits	3,585	3,334	38.4	3,624	3,522	39.1	1,807	1,071	29.6
Mushrooms	60	49	0.6	60	56	0.6	45	27	0.7
Confectioneries	176	284	1.9	160	250	1.7	127	280	2.1
Beverages	3,410	2,488	36.5	3,282	2,513	35.4	1,768	1,176	29.0
Green tea	3,207	2,503	34.3	3,074	2,499	33.2	1,602	1,177	26.3
Energy adjustment (residual method)									
Total food	8,998	3,764		8,748	4,014		6,051	2,057	
Cereals	376	424	4.2	482	826	5.5	101	179	1.7
Potatoes	236	160	2.6	230	163	2.6	247	123	4.1
Nuts and seeds	86	212	1.0	69	194	0.8	56	104	0.9
Vegetables	1,365	737	15.2	1,305	672	14.9	1,816	625	30.0

Fruits	3,267	2,050	36.3	3,208	2,062	36.7	1,752	963	29.0
Mushrooms	58	45	0.6	56	49	0.6	44	27	0.7
Confectioneries	186	334	2.1	160	261	1.8	125	307	2.1
Beverages	3,393	2,413	37.7	3,272	2,532	37.4	1,774	1,193	29.3
Green tea	3,194	2,452	35.5	3,063	2,529	35.0	1,611	1,203	26.6
TRAP, μmol TE/d									
Crude values									
Total food	6,400	4,110		6,199	4,121		3,510	1,857	
Cereals	50	50	0.8	50	54	0.8	20	25	0.6
Potatoes	19	16	0.3	19	19	0.3	19	11	0.5
Nuts and seeds	1	2	0.0	1	3	0.0	5	12	0.1
Vegetables	563	393	8.8	535	410	8.6	551	185	15.7
Fruits	881	856	13.8	892	908	14.4	419	237	11.9
Mushrooms	131	108	2.0	130	123	2.1	98	58	2.8
Confectioneries	29	46	0.5	26	41	0.4	15	32	0.4
Beverages	4,725	3,613	73.8	4,544	3,608	73.3	2,378	1,712	67.7
Green tea	4,618	3,603	72.2	4,425	3,598	71.4	2,307	1,695	65.7
Energy adjustment (residual method)									
Total food	6,299	3,711		6,064	3,884		3,508	1,872	
Cereals	48	46	0.8	50	54	0.8	20	25	0.6
Potatoes	19	15	0.3	18	15	0.3	19	11	0.5
Nuts and seeds	1	2	0.0	1	2	0.0	5	13	0.1
Vegetables	543	331	8.6	493	262	8.1	546	172	15.6

Fruits	798	507	12.7	783	502	12.9	409	220	11.7
Mushrooms	127	98	2.0	121	108	2.0	97	59	2.8
Confectioneries	29	48	0.5	25	39	0.4	15	30	0.4
Beverages	4,710	3,557	74.8	4,519	3,606	74.5	2,389	1,744	68.1
Green tea	4,599	3,532	73.0	4,409	3,642	72.7	2,320	1,734	66.1

FFQ, food frequency questionnaire; DR, dietary record; SD, standard deviation; TE, trolox quivalent; FRAP, ferric reducing-antioxidant power; ORAC, oxygen radical absorbance capacity; TRAP, total radical-trapping antioxidant parameter.

[a] FFQ_R was administered 1 year after and before FFQ_V.

[b] FFQ_V was administered 1 year after completion of the DRs.

[c] DR was collected over a 1-year period.
